# Navigating patients’ refusal of information in clinical practice - a clinical scenario

**DOI:** 10.1007/s11019-026-10356-0

**Published:** 2026-05-02

**Authors:** Jenny Lindberg

**Affiliations:** 1 Department of Clinical Sciences Lund, Medical Ethics, Lund University, Lund, Sweden; 2 Department of Nephrology, Skåne University Hospital, Malmö, Sweden

**Keywords:** The right not to know, Self-determination, Authenticity, Decision-making, End-of-life care

## Abstract

The right of patients to decline information about their health, prognosis, and available treatment options is a salient principle in both domestic law and international declarations and conventions. This right may be considered either unnegotiable or subject to certain terms and conditions. While respecting this right may seem straightforward, doing so in clinical practice can be challenging. This article uses a realistic hypothetical scenario to examine the epistemic, moral, and practical challenges that can arise, particularly with patients nearing the end of life. These challenges include how and when to honor the right and its potential conflict with moral values such as self-determination, authenticity, and avoiding harm to oneself and others. The end of life exacerbates these issues because of the irretrievability of decisions and the successive reduction of possible courses of action, as well as potentially changing preferences. These potential conflicts of values deserve further attention and must be considered when deciding whether to honor a patient’s wish not to know.

## Introduction

Healthcare professionals are often expected to respect patients’ wishes not to know about their disease, prognosis, or treatment. According to a stronger formulation, a right not to know for patients has been advocated. This right is enshrined in domestic legislation, as well as in international declarations and conventions (WMA 1981; Council of Europe 1997; UNESCO 1997; Socialdepartementet 2014). Although respecting this right^1^ may first seem straightforward, it is not always clear how to understand and respect it in real-life situations. This paper explores these uncertainties through the lens of a complex yet realistic clinical scenario involving a patient with chronic, deteriorating diseases and an accumulating symptom burden, where several decisions must be made progressively throughout the patient’s journey towards the end of life. The paper is structured as follows: First, a background is provided on the right not to know in medical ethics. After that, a hypothetical realistic scenario is presented to illustrate some of the challenges of respecting a patient’s wish not to know when approaching the end of life in a nephrology care setting. The scenario is then analyzed from epistemic, normative, and practical angles to illustrate the difficulties of respecting the right not to know in clinical practice. The paper concludes with a commentary on the implications of the analysis.

## Background

Healthcare largely has moved away from the paternalistic paradigm of the past (Buchanan 1978). Nowadays, respect for patient autonomy is central in modern Western healthcare, at least in principle (Coggon and Miola 2011; Beauchamp and Childress 2019). While patient self-determination does not encompass the right to freely choose treatment options, it involves a possibility for patients to accept or refuse the interventions and care that are offered to them (Faden and Beauchamp 1986). Healthcare has further been urged to follow the ideal of patient- or person-centered care (Mead and Bower 2000; Leplege et al. 2007; Ekman et al. 2011) and strive for shared decision-making (Charles et al. 1997; Elwyn et al. 2012; Stiggelbout et al. 2015; Engelsma 2024). This shift incentivizes healthcare professionals to engage patients in decision-making processes.

One of the fundamental prerequisites for patients to meaningfully exercise their self-determination and actively participate in their own care is that they are appropriately informed. However, what information is appropriate has not been uniformly clarified. What has been recommended is broadly that the consent or dissent should be adequately (Murray et al. 2007; Cocanour 2017) or *substantially* informed, and that the information should include those things that a reasonable person would need or want in order to consent or dissent to treatment (Faden and Beauchamp 1986). This, in turn, has been taken to include relevant information concerning the nature of interventions and the foreseeable outcomes of these interventions (Faden and Beauchamp 1986).

Respect for patients’ self-determination has been understood to encompass a respect for patients’ wishes *not* to receive certain (or any available) information about their life and health, that is, their right not to know (Herring and Foster 2012; Laurie 2014). This right has been debated in contexts where much information is available as a result of genetic analysis, but where patients only wish to receive part of it (Hayry and Takala 2001; Andorno 2004; Knoppers and Dam 2011; Juth 2014). It has also been discussed in other areas, such as when HIV test results are to be shared (Andorno 2016; Youngs and Simmonds 2016) or when individuals are to be informed about their risk of developing a neuropsychiatric disorder (Bortolotti and Widdows 2011). While many patients want information about the risk of developing a disease, its consequences, and potential future life conditions, not all patients share this wish.

There may be several reasons for respecting patients’ wishes not to know, and these have been previously discussed in the literature. One reason is to honor patients’ privacy and avoid forcing information on them, in analogy with not imposing a bodily intervention on them (Laurie 2014). Another reason to respect the wish not to know could be to avoid harming patients by forcing them to confront burdensome information (Colloca and Miller 2011). A third reason could be that respecting the patient’s wish not to know can be considered an application of the general call to respect patients’ self-determination (Husted 2014) and the related idea that (more or complete) information will not always help patients to make decisions that are in line with their own preferences (Herring and Foster 2012; Rees 2023). This idea has been used to justify withholding *some* information, but not necessarily all of it, in general and in situations where patients express a desire to not receive it (Rees 2023). While there are reasons to respect a patient’s wish not to know, it has also been claimed that there might be overriding reasons to not respect it, at least in certain contexts (Harris and Keywood 2001; Malpas 2005; Bullock 2015). Even when speaking with patients who are severely ill or dying, there has historically been a tendency to respect their wish to not know (Saunders and Sykes 1993; Saunders et al. 2006). At the same time, there is a call for compassionate transparency with dying patients (Loprinzi et al. 2010) as well as an underlying ethos of palliative care suggesting that patients should be given the means to affront and accept death when it comes (Sulmasy 2013).

Clearly, there is a potential tension between the right to be informed and the right not to be informed about relevant healthcare matters, including those related to palliative care. In a recent article, Joar Björk addresses this issue, shedding light on it from the perspective of the palliative care ethos. According to Björk, this ethos encompasses helping patients accept their situation, striving for a good death, planning ahead, and building relationships, among other things (Björk 2025). Reaching these goals will encounter challenges if patients remain uninformed about their disease status, prognosis, and treatment options. While the palliative care ethos may provide guidance for ethically acceptable solutions to arising problems, it must also, according to Björk, be critically analyzed and compared with claims from mainstream bioethics (Björk 2025). The present paper approaches the question from a standpoint that could be considered mainstream bioethical, especially with a focus on moral values. Questions arise: Should a patient’s wish to not receive information at any point in life or in the disease trajectory be respected? What other considerations might arise that may conflict with respecting the right not to know?

Most patients probably prefer that their physician, when relevant, delivers bad news about their illness and impending death. Nephrology patients typically consider it important to play an active role in end-of-life planning to ensure that their personal priorities and preferences are respected (Davison 2006; Sellars et al. 2018; Ladin et al. 2021). But there is a wide range of preferences about involvement in advance decision making among patients with chronic kidney disease (Orsino et al. 2003; Phillips et al. 2018). Some patients with chronic kidney disease who are approaching death maintain an unrealistic hope of surviving, which might lead them to overestimate their life expectancy and avoid end-of-life conversations (Calvin 2004; O’Halloran et al. 2018) or at least postpone such conversations until their condition deteriorates (Phillips et al. 2018). Patients might not always fully grasp the expected effects of the care they receive^2^. This can compromise the informed consent process by causing patients to underestimate or overestimate the risks and benefits of certain treatments. Information about being at the end of life and the consideration of life-prolonging care as futile can be difficult for patients to accept. It comes at a time when patients may be particularly vulnerable due to their disease and the existential concerns associated with it, and it might be difficult to cope with all the information at that time.

When facing increased frailty and worsening symptoms, even patients who have chosen not to know may suspect that all is not going well and that the end of life is approaching. This could elicit various reactions, and it will not always be easy to tell whether patients understand anyway where their disease is taking them. Complicating matters further, there is always some uncertainty regarding prognosis and predicting time to death, although attempts have been made to do so in patients with chronic kidney disease (Moss et al. 2008; Cohen et al. 2010; Da Silva Gane et al. 2013; Schmidt et al. 2019). This uncertainty may cause healthcare professionals to be unsure about the timing of end-of-life conversations (O’Halloran et al. 2018). In the early days of hospice care, preserving the patient’s hope and avoiding despair in the face of impending death were seen as important goals (Kübler-Ross 1975, 1997). Wanting to maintain the patient’s hope could motivate healthcare professionals to withhold information, whether it is done on the patient’s request or their own initiative. There might, of course, be other explanations why they, at least temporarily, withhold certain information (Axelsson et al. 2019).

More recently, a patient-centered approach to sharing information about prognosis has been advocated. This approach recommends starting the conversation with the patient by asking, “How much do you want to know?” (Back and Arnold 2006). However, patients may find this question difficult to answer because their response will likely depend on the type of information to be provided, which they do not yet know anything about. The information that needs to be communicated often comes down to highly specific, individualized details. Asking this question demonstrates a willingness to adapt the content and timing of information to the needs and wishes of individual patients, which is in line with what has been suggested for shared decision-making (Austin et al. 2015; Rietjens et al. 2017). This includes respecting a patient’s wish to not be informed. However, balancing the ideal of informing patients with their wish not to be informed can place strain on healthcare professionals when striving to act in patients’ best interests. (Björk 2024). Withholding information at a patient’s request may present several other challenges, as will be demonstrated by studying how this plays out in practice.

### From principle to practice: the right not to know in a real-life scenario

As with other ethical norms and principles in healthcare - such as respecting patient autonomy, beneficence, nonmaleficence, and justice - a principle that tells us not to inform a patient should also provide ethically acceptable guidance in concrete cases. The fictitious, yet realistic case of Martha described below will serve as a starting point to examine the principle, what must be considered when applying it, and its potential consequences. All the features of this scenario are common in a nephrology care setting and are easily recognizable from my own clinical experience. Martha could undoubtedly be a patient at any nephrology clinic, whether in Sweden, where I work, or elsewhere with a similar healthcare system. Consider the following case:

Martha is 76 years old. She has diabetes mellitus, congestive heart failure, and kidney failure. She has been on hemodialysis for five years. She experiences severe pain due to peripheral arterial disease with chronic ischemia and polyneuropathy. Her medications include painkillers that only relieve, not eliminate, the pain. She also suffers from nausea and fatigue and has lost 10 pounds in the last six months. She no longer spends time at her summer house and is too tired to do much outside activity between dialysis sessions. Martha lives alone, but her adult sons and their families visit her on weekends. She does not want to bother them with details about her health. She has been in and out of the hospital several times in the past six months due to infections. The healthcare team at the dialysis clinic caring for Martha has good reasons to believe she does not have much time left to live, at a maximum 12 months. They believe it would benefit her to participate in advance care planning so that she can express her preferences for the time she has left. However, as soon as they try to open a discussion about her disease, prognosis, or treatment options, Martha firmly expresses that she does not want any information about her health. She also tells the healthcare professionals that she does not want to make any decisions about treatment options, either present or future. In fact, she wants them to make all the decisions. Despite several attempts to discuss the situation and the options with Martha, she does not allow it, exclaiming, “I don’t want to know anything! You decide what to do!” When the healthcare professionals try to understand her reasoning, she repeats that she does not want to speak about the issue.

This case helps us shift our focus from an abstract, general level, where adhering to the principle may seem straightforward, to the complexities of real-life clinical situations, where the challenges of doing so become more apparent. These challenges are both of an epistemic and moral nature, but practical issues must also be considered, as explained below. Epistemic uncertainties and practical issues overlap with moral considerations about how to comprehensively weigh the identified values, which conflict with one another, to reach normative conclusions.

#### Uncertainty regarding Martha’s underlying reasons or motives

One key question concerns Martha’s underlying reasons, or motives, for not wanting information. Uncertainty regarding these things has been recognized by healthcare professionals taking care of dying patients who choose not to receive information (Björk 2024). There are several possibilities. Martha could sense that there is not much time left in life but does still not want to talk about it or plan for it because it would be too painful for her, causing distress. This has been addressed in the literature (Chibnall et al. 2002). She could therefore not want to receive the information at all or want to postpone it. She might also be ambivalent regarding her wish to know or not to know, which is one possible reaction as one approaches the end of life (Sand et al. 2009). She could engage in self-deception such as denial or wishing for an ultimate (and unrealistic) cure, which is also common when approaching death (Kübler-Ross 1997; Sand et al. 2009). It is possible that Martha’s motives are based on misunderstandings regarding her condition, prognosis, treatment options, and discussions with her healthcare providers. Of course, she may also have expressed a rational, stable, well-considered, and enduring wish not to receive information. Or she may have several different motives of both rational and emotional nature that cause her to not want to know about her illness, treatment, and proximity to death.

Why Martha desires to remain ignorant is a key issue because the variety of possible underlying motives raises the question of what one is really respecting when one respects her desire not to know. On one account, Martha’s wishes should be respected regardless of her motives. She possesses - as it is here assumed – the degree of decision-making capacity we normally respect, and normally patients need not to provide a rational explanation nor provide one that healthcare professionals accept. There might therefore be a tension between the legal and ethical perspective, since there is normally no legal duty to explain the motive for the refusal of information. This includes cases where the resulting decision, from an external perspective, seems detrimental to patients’ welfare (Savulescu 2007). However, in a moral sense, there appears to be a relevant difference between a wish based on false beliefs or fear of unknown dangers, and an autonomous informed wish not to know. Martha might for example think that she will die tomorrow, or that she will die in unbearable pain, or that there are other distressing facts that she wants to shield herself from, while the truth is that there might be expectations of a whole year left to live and the palliative care team will be able to manage her symptoms at the end of life. Furthermore, there is no guarantee that Martha’s wish is based on her personal values and preferences since she may not know anything beyond the fact that there is relevant information that she could receive.

If Martha’s wish is based on a misunderstanding, healthcare professionals should arguably at some point attempt to correct this misunderstanding. This could, for example, help her understand how much time she has left to live, and the treatment options available for her symptoms. It could also assist her in overcoming her fears of death (Cozzolino et al. 2014). Such a clarification could be provided without demanding a rational explanation for not wanting to know. The problem, however, is that even investigating these issues would likely reveal the kind of information that Martha has declined to receive.

#### Uncertainty regarding the scope of what Martha does not want to know

As we have seen, understanding Martha’s motives is important for several reasons. In addition to the reasons mentioned above, the motive can be relevant to determine the scope of information she does not want to receive. One way to approach this is to exclude all information. However, this does not necessarily align with what she wants. Another possibility is that the healthcare professional interprets Martha’s wishes and excludes information that the professional perceives as sensitive for Martha. This assumes that this kind of information matters most, but this may not align with Martha’s view. In either case, there is no clear path to follow regarding the type and amount of information to provide. Withholding all information or singling out certain clinical information can be hazardous. With only a vague understanding of the reasons for her denial, it is difficult to determine the extent to which Martha would rather not receive information. For example, Martha may want to know that she has the option to reduce her dialysis frequency if she feels that the sessions are burdensome, while not wanting information about her being close to death. Likewise, she may want to know about effective symptom relief without wanting to know the details of all the expected symptoms. She may also want to know about home care options, even if the end-of-life situation is not fully explained to her. Therefore, despite saying that she does not want to know anything, Martha may want to know certain things. Her ideas and the healthcare team’s ideas of what should be included or excluded may differ, and this distinction is impossible to make without asking Martha. To respect Martha’s wish not to know, healthcare professionals must know how to do so, understanding what is and what is not included in her wishes.

#### Uncertainty regarding the duration of the wish not to know

There might also be uncertainty as to the temporal aspect of her wish not to know, as preferences might likely change (Björk 2024). It is not possible to know whether Martha’s informational preference changes without asking her, something healthcare professionals should avoid if her right to privacy is to be honored. Martha’s motives may be based on momentary attitudes, raising the question of whether her current desire to not be informed should be taken at face value as an ongoing desire. There is uncertainty about whether healthcare professionals should require Martha to reiterate her wish not to know multiple times throughout the progression of her disease to ensure it remains an ongoing wish. Respecting her initial statement could prevent them from honoring her potential future desire to know certain things. They could feel obligated to at least seek some indication of her current desire to know or not know. However, repeatedly asking her if she wants information could cause her to accept it because of the way the question is worded or because she is tired of being asked. In short, there is no simple or safe way to respect Martha’s initial wish not to know, nor to ensure that her actual wish is fulfilled over time.

#### An autonomous wish to opt out of autonomy?

The principle of autonomy or patient self-determination, as understood here, refers to the obligation of healthcare professionals to protect and respect patients’ right to make informed and voluntary decisions about their own care (Beauchamp and Childress 2019). Following this principle would arguably mean also respecting the (autonomous) wish of Martha not to receive information (Andorno 2004; Herring and Foster 2012; Husted 2014).

Normally, healthcare professionals respect patients who decline certain treatment without first investigating whether they choose rationally and according to what they really want. However, in cases where the treatment is of fundamental importance to restore health and the patient seems not to have understood this, it might be morally acceptable - or even required - to repeat the information to try to ensure that the patient understands the benefits of the intended treatment in a way that the patient also understands the consequences of declining it. In the case of Martha, if her wish not to know is respected, she will not have any updated information whatsoever (or so it is assumed) about the disease, prognosis and potential treatments, which makes even the knowledge that there are decisions to make unavailable to her. Since Martha is unaware of the nature of the information she is declining, it seems impossible for her to properly assess the risks of not having it. Her ignorance undermines the basis of informed refusal. Furthermore, the healthcare professional is not in the best position to judge whether the risks of knowing or not knowing are acceptable to Martha. Martha is the best, or only, judge of that, since she knows the importance of the different risks in relation to her personal priorities in life.

What is at stake for Martha is not only the opportunity to make autonomous decisions here and now. Her decision to give healthcare professionals *carte blanche* for any future decision will keep her in the dark about decisions that are still to come. Such decisions can concern a wide variety of situations that may arise during her remaining time in life. It is not just about knowing the risk of a disease, such as receiving results from genetic testing. In this case, it is about the expected continuous and impending deterioration of her health. This deterioration will lead to an increasing symptom burden, requiring medical interventions for which Martha has not provided - and cannot provide - informed consent.

Personalized decisions may be particularly important at the end of life. When facing death, the remaining time arguably becomes more important regarding how we live it and what we make of it. Also, as Martha is approaching the end of life, the range of choices she can make may gradually shrink, resulting in irreversible or irretrievable decisions. For this reason, the stakes of current decisions may be higher than at other times in life when more time remains and a wider range of possibilities exists in the future. Respecting Martha’s wish not to know comes with a risk to close off future opportunities for her to make important decisions for her remaining life, and she will not know what kinds of decisions she is thereby giving up.

Failing to provide Martha with important information regarding her life could give rise to moral and epistemic asymmetries between her and the healthcare professionals. Shielding herself from such information - and even more so, demanding to be shielded from it indefinitely - might be a problem for her ability to shape her remaining life authentically. This means that Martha will not be able to be true to her values, a significant component of authenticity (Taylor 1991).^3^ A lack of important information about herself may also lead to a reduced capacity to pursue what is good for herself and others (Hill 1991; Haksar 1998; Garnett 2023). Abdicating from knowing about and taking control of her own life can be considered a deferent attitude that might be detrimental to Martha whenever it occurs in life as a form of lacking self-respect (Hill 1991) but potentially more so at the end of life, when priorities may change in light of the limited remaining time. It is unclear to what extent Martha can be held accountable for how her life develops when she chooses to withdraw from decision-making and leaves relevant information and decisions in the hands of others. However, she can reasonably to some extent be held accountable for failing to engage in moral reflection and dialogue with others, which would enable her to shape the rest of her life authentically (Taylor 1991). Others will have to take the lead and decide for her, which makes her vulnerable to domination and to living a life that serves the interests of others instead of her own. This could be detrimental to Martha, even if she herself has chosen this situation.

One could argue that Martha might be authentic in her desire to not know. It could, for example, be “like Martha” not to engage in planning and safeguarding her interests. However, it is difficult to draw normative conclusions from the fact that such desire could be authentic. Furthermore, it appears to be a self-defeating form of authenticity. While it may stand for the expression of some kind of liberty, letting go of all moral reflection and social responsibility does not align with the conception of authenticity as previously explained.

#### Negative impact on Martha’s welfare

Understanding Martha’s motives is also necessary to determine how Martha will be affected by receiving or not receiving information. As shown above, respecting Martha’s wish not to be informed will prevent her from participating in current and future treatment decisions. It will not be possible to obtain her informed consent for interventions that, from the healthcare professionals’ perspective, would benefit her. Furthermore, Martha will not be able to exert influence over her care, which is an important prerequisite for avoiding harm. In other words, healthcare professionals may be prevented from providing good care since they are unable to benefit Martha in the way she is personally best cared for.

It will be difficult to adhere to treatment plans without explaining their rationale to Martha. And lack of adherence could negatively impact Martha. For instance, she might accept more extensive pain relief if healthcare professionals could assure her that she will not become addicted to strong medications, given her limited life expectancy. It would be difficult for a healthcare professional to prescribe strong medications to her and expect her to administer them herself (which is often the case in early phases of palliative care) without first explaining their purpose. If there is a consensus between Martha and the healthcare professionals on avoiding overly aggressive care at the end of life, Martha could be given the option to avoid going to the emergency room as she deteriorates and receive home care instead. She could also aim to reduce or stop dialysis if she would rather stay at home with her family than be in the hospital. She could prioritize spending time at her summer house, which she currently lacks the time or energy to visit. She could also choose where to die, something that dying people usually appreciate.

Furthermore, knowing what lies ahead in the coming months will make it easier to address the psychosocial and existential distress that might arise. This will allow the healthcare professionals to support Martha throughout her disease trajectory and help her maintain realistic hopes for the future. Protecting her from painful information now may cause greater harm later due to a lack of psychosocial preparation.

#### Negative impact on others

If Martha is not informed about matters concerning her health and illness, the consequences may affect others as well. Family members may have different needs regarding information that Martha has refused. Different family members may further need different information. In general, family members may want information about a patient being at the end of life for several reasons. Martha’s refusal to receive information may primarily affect her adult sons. If they receive information that Martha declined, they may find themselves in a difficult situation, having to decide how to approach the topic with their mother. Knowing that she refused the information they received from healthcare professionals could pressure them to hide things from Martha. This would imply that they would have to approach her dishonestly and potentially mislead her. The situation becomes more complex if they inadvertently or unconsciously reveal relevant information to Martha, such as the fact that she is approaching the end of her life.

The right to not know in healthcare does not imply the right to absolute ignorance. Such a right would be difficult to maintain in society because information could be exchanged through personal relationships and other means that are more difficult to control. For this reason, as debated in the literature, the right not to know has primarily been limited to the relationship between healthcare professionals and patients (Davies and Savulescu 2021). This means that family members are not legally obligated to protect patient-specific rights since they do not have a caregiving relationship with Martha. Therefore, if family members inadvertently inform Martha that she is dying, they are not disrespecting her legal right not to know. However, there may be moral reasons to withhold information in certain situations, including in personal relationships. Nevertheless, uncertainty remains regarding how the moral claim on Martha’s sons should be interpreted - specifically, whether the sons could, from a moral point of view, reveal information about her condition to her, at least to some extent. The uncertainty regarding this moral assessment might put further strain on Martha’s sons.

Where the risk of harming others is significant, the right not to know must be contrasted to the harm caused to both significant others, healthcare professionals and to the healthcare system in general (Davies 2020). Ensuring that the family will not be harmed by a lack of opportunity to prepare for her death may require that Martha knows that she is dying and what lies ahead. This is at least how it is usually assumed in a Western cultural context, as opposed to some more family-oriented contexts, where bad news about the disease and prognosis could be expected to be withheld from the patient but provided to the family.

Besides Martha’s family, her wish to stay uninformed affects the professional team caring for her. This team may consist of nephrologists and other physicians, registered nurses, nurses, social workers, physical therapists, occupational therapists, and others. Requesting that information be withheld from Martha would force all these healthcare professionals to do so in all situations, not just at one point in time. The requirement not to inform Martha will thus affect many people indefinitely. This attitude of intransparency toward Martha differs from what is normally expected of healthcare professionals. Not only will they be forced to act non-transparently toward Martha, but to ensure that Martha will not be accidentally informed will also require significant coordination efforts. Martha will have to be treated differently, which could cause confusion and put healthcare professionals at risk of unintentionally breaking their promise not to disclose information she does not want to receive. They could even be forced to use lies to conceal the reasons for her increasing symptoms and frailty - something that would otherwise be unacceptable. The silence imposed on healthcare professionals may distract them and cause them to focus on not unintentionally providing Martha with information, instead of focusing on their task of treating her adequately. Interprofessional communication with Martha present in the room will be hindered, which could reduce safety in healthcare situations. This will be even more problematic for healthcare professionals who meet Martha several times and develop a personal relationship with her.

Respecting Martha’s wish may also have consequences for healthcare in a broader sense. Lack of adherence, because of lack of information, has consequences for Martha in the long run as explained above, but might also have consequences for others. Informing Martha could make her in turn share more information regarding her symptoms and this could help the healthcare professionals to fulfill their task of offering good symptom relief, which they are hindered from through not being able to have that conversation with Martha. A lack of adherence might lead to greater healthcare costs, with impact on other patients. Avoiding end-of-life conversations may furthermore cause more aggressive care towards the end of life, carrying with it a worse quality of life and less possibilities to adjust to what life will be like (Wright et al. 2008). There is a risk that the decision to reduce or stop futile treatment, such as dialysis, will be postponed, which could result in financial costs for healthcare, displacement effects on other patients, and increased suffering for Martha.

By refusing to receive information about her condition, Martha is essentially forcing healthcare professionals to make decisions in which she is entitled to participate. Taking full responsibility for the decisions may cause healthcare professionals to ensure they are right in other ways. For example, they may wait for Martha to deteriorate further before withdrawing dialysis to confirm its futility. Even though Martha has the right to make decisions about her care, a right that is not abolished just because she chooses to refuse information, these decisions will be highly hypothetical without informing her of the reasons for the decisions and the consequences of the different alternatives.

Responsibility for deciding on matters other than Martha’s care may transfer to family members, who will effectively act as surrogate decision-makers for a person with decision-making capacity who, due to lack of information, cannot participate in decision-making. Informing Martha’s sons without informing Martha would burden them with the responsibility of making decisions about her life and belongings, in which she should have participated. It is not uncommon for family members to make such decisions, since it occurs whenever the patient is incapable of participating in them. When they assume this role with patients lacking decision-making capacity, it may be the only way to protect patients’ interests and honor their preferences. This ideal will not be perfectly realized, but given the circumstances, it is the best alternative. In Martha’s case, however, others will have to guess what she wants, even though she has the capacity to decide and communicate. Although Martha has chosen to defer decision-making to others, it will put a strain on them because the ideal is to decide according to the patient’s wishes and preferences. Disconnecting this ideal from surrogate decision-making and accepting choices that may not align with Martha’s preferences, despite the possibility of inquiring about them, is an unfortunate step taken to reduce the strain.

#### Conflicts with the ideal of advance care planning and palliative care

In addition to the epistemic, practical, and moral uncertainties surrounding Martha’s right to not know, doing so may conflict with key healthcare ideals and expectations regarding how healthcare professionals should behave toward patients. Since patients like Martha are relatively rare, healthcare professionals may be unprepared and lack guidance on how to handle the situation. They might feel the duty to engage Martha in decisions regarding her disease and treatment, at the same time as they feel the duty to respect her wish not to be engaged. In nephrology, the ideal approach toward the end of life is to engage patients and their families in advance care planning (Davison 2006, 2010; Amro et al. 2016; Goff et al. 2019). Respecting a patient’s wish not to know when approaching the end of life conflicts with several ideals of advance care planning and palliative care, including planning ahead, following the patient’s values and preferences, and adapting to changes in priorities over time (Houben et al. 2014; Rietjens et al. 2017, Radbruch et al. 2020; Björk 2025). Martha’s involvement in healthcare decisions requires that she is informed about what is relevant to her. It presupposes a dialogue between healthcare professionals, the patient, and the patient’s family. However, this process cannot take place without Martha. As individuals approach the end of life, topics beyond medical facts arise for discussion between healthcare professionals and patients. The focus may shift to other issues that are important to the patient (Björk 2025). These include psychosocial and existential issues, which are also part of end-of-life conversations with palliative care patients (Kübler-Ross 1975, 1997; Saunders and Sykes 1993; Saunders et al. 2006; Loprinzi et al. 2010). Open and straightforward communication about Martha’s condition could help her come to terms with her imminent death, which she would be denied if her wish to remain uninformed is honored.

### Where does this all leave us?

By now, it should be clear that honoring a patient’s wish not to know can be complicated. Considering the whole situation - the epistemic, practical, and moral challenges involved, as well as the conflict with the ideals to which healthcare should normally aspire - respecting Martha’s decision not to be informed is anything but straightforward. There might be exceptions, of course; situations in which one should respect a patient’s wish not to be informed because the reasons for doing so are significant and uncontroversial. But there will be many situations where this is not the case.

Respecting the right not to know in a case like Martha’s carries uncertainties and risks, and it is, as has been shown above, not obvious what precisely to respect (a rational wish, an emotional response, a reaction based on misunderstandings, etc.) nor for how long. Even Martha herself might not completely comprehend her underlying motives for not wanting information. At the same time, disregarding or downplaying Martha’s wishes should not be taken lightly, as doing so could violate her privacy and right to self-determination. It could also hinder her ability to protect herself from harm and distress. In other words, it could manifest the kind of paternalism that has been abandoned in modern times. Therefore, honoring or not a patient’s wish not to know is a highly complex decision that requires consideration of many factors.

Do not many of the challenges mentioned above emerge whenever one is expected to respect someone’s decisions? In one sense, the answer is yes because it boils down to understanding what one is supposed to respect, and the consequences of doing so. Uncertainty about what patients like Martha want and do not want is something healthcare will always have to deal with. However, the problem runs deeper when the decision concerns not wanting any information or further discussion about the matter. In that case there is little or no possibility to make inquiries, as this communication could reveal or remind her of things she does not want to know. If a patient does not receive any information, there will be more than just misunderstanding. There will also be a lack of knowledge necessary for the patient to understand what is at stake, and again, there will be no opportunity to clarify things.

The challenges mentioned above raise the question of whether healthcare professionals should have any role in helping Martha to be a moral agent who is accountable for her own life, or if they are responsible for helping her live her life authentically. This question will be left open for now. Acting to help Martha make authentic choices would potentially safeguard her future authentic decision-making, thus serving the value of autonomy (Sjöstrand et al. 2013). Information about her health and life situation is certainly crucial for Martha to plan for her remaining time in life. This is true at any stage of life, but knowing she is near death could make her more likely to want to settle her life and plan for what she would like to accomplish before death. This might include fulfilling obligations towards the family and significant others.

One could ask whether it would be possible to ensure end-of-life care in accordance with Martha’s preferences and to serve her interests without informing her directly. This raises the question of whether this could be done through hypothetical conversations, such as asking: “What would you want if this happened?” or similar questions. Another possibility is using nudges to help her make good decisions despite her lack of complete knowledge about the situation. However, these strategies are not likely to succeed. First, they would require Martha to be provided with some information, which would go against her wish not to talk about it. Second, the discussions would be hypothetical and distant. Even if the decisions align with Martha’s preferences, they would be based on her uninformed survival expectancy. This expectancy could be significantly shorter or longer than how healthcare professionals judge the situation. Adopting that perspective would not help determine Martha’s actual preferences, as they could easily change depending on where on the road towards the end of life the healthcare professionals have reasons to believe that she is (Björk 2025) (assuming that healthcare professionals have accurate prognostic skills). The only way Martha can understand and make personal decisions is if she is provided with information straightaway. In other words, there are no shortcuts that would enable Martha to live her remaining life authentically.

We have seen how a hypothetical clinical scenario can highlight the potential complications of respecting patients’ right to not know. When applied to this scenario, it becomes clear that honoring this right is not straightforward, even as a prima facie principle. There are epistemic obstacles that prevent an optimal moral assessment of the different options, and of situations in which this principle must give way to others. Furthermore, as we have seen, there are several epistemic, moral, and practical uncertainties about how to honor this principle in clinical practice. The moral implications include the impact on future autonomy and the ability to make authentic end-of-life choices, potential harm to the patient and others, and the problematic double standard of being dishonest with the patient while being expected to be transparent in general and with other patients. These challenges should be considered in future discussions about the role and responsibility of healthcare professionals toward their patients regarding information sharing.

## Footnotes

1 In this article, the term “right” will be used broadly and noncommittally. This usage does not imply endorsement of any particular theory of rights nor presuppose the existence of such rights.

2 Therapeutic misconception, which is analogous to this misestimation, is a well-analyzed issue that will not be discussed further here, since the focus of this article is palliative care, not research.

3 According to Charles Taylor, not all subjective values are equally valid. Instead, authenticity requires the subject to choose what is worthwhile - things that matter in an objective sense. This condition requires moral reflection and social responsibility, encouraging dialogue with and recognition from others. Another definition includes being true to oneself in a way that enables procuring one’s own benefit, including notions of sincerity with oneself (Varga 2023).
